# Nalmefene Mitigates Opioid‐Induced Nausea and Vomiting in Postoperative Analgesia but Not Resting Pain

**DOI:** 10.1155/prm/5531157

**Published:** 2026-05-25

**Authors:** Jiaojiao Yang, Baoling Zhang, Lili Qiu, Qiuting Zeng, Jue Xie, Jie Sun

**Affiliations:** ^1^ Anesthesiology, Surgery and Pain Management Department, Zhongda Hospital, School of Medicine, Southeast University, Nanjing, Jiangsu, China, seu.edu.bd

**Keywords:** nalmefene, nausea and vomiting, patient-controlled analgesia, postoperative analgesia

## Abstract

**Background:**

Patient‐controlled analgesia (PCA) pumps have emerged as the prevalent modality for managing postoperative pain. Opioids, although widely utilized as analgesic agents in these pumps, are often accompanied by undesirable side effects that can compromise patient comfort and hinder the widespread adoption of PCA therapy.

**Methods:**

We conducted a retrospective study involving 392 patients undergoing lumbar spine surgery who were prescribed PCA pumps for pain relief. Over the initial 1–3 days postsurgery, comprehensive data encompassing resting and activity‐related pain levels, Ramsay sedation scores, activity status, and flatus passage were carefully recorded and analyzed.

**Results:**

Interestingly, patients who received a supplemental dose of nalmefene in their PCA pumps exhibited a notable increase in resting pain intensity on the second postoperative day. However, the incidence of postoperative nausea and vomiting (PONV) was notably reduced (15.5% in the sufentanil + nalmefene group vs 25.5% in the sufentanil group). Notably, no statistically significant variations were discerned between the two groups in Ramsay sedation scores, postoperative activity capabilities, flatus passage, or inflammatory biomarker levels.

**Conclusion:**

The integration of nalmefene into PCA pumps presents a promising strategy for mitigating the occurrence of PONV, albeit with the caveat of potentially compromising opioid‐mediated analgesia, necessitating further research and exploration. The delicate balance between enhancing patient comfort and preserving effective pain control remains a critical area of investigation in the field of postoperative pain management.

Pain is a ubiquitous experience among surgical patients, particularly during the initial postoperative period. Patient‐controlled analgesia (PCA) represents a widely used technique that empowers patients to adjust the dosage of analgesics in accordance with their individual pain levels, fostering a more personalized approach to pain management [1]. Opioids, notably sufentanil, are routinely administered in the perioperative phase to alleviate pain; however, they are also known for their significant adverse effects when utilized for postoperative analgesia. These effects encompass several undesirable symptoms, including diminished respiratory drive, coughing suppression, urinary retention, nausea, vomiting, and constipation. Notably, postoperative nausea and vomiting (PONV) are particularly prevalent in the postoperative setting, causing not only direct discomfort but also contributing to adverse surgical outcomes, including elevated arterial pressure, aspiration risks, postoperative bleeding, and suture leaks [2]. In the context of the enhanced recovery after surgery (ERAS) pathway tailored for spine surgery patients, achieving effective analgesia while minimizing adverse effects has emerged as a cornerstone of ERAS guidelines. In pursuit of this goal, various strategies have been devised to mitigate the adverse reactions associated with opioid use in analgesic pumps, including the application of topical nonsteroidal anti‐inflammatory drugs around the incision site, neuron blocks, and the employment of dexmedetomidine [3–6].

Nalmefene, an antagonist of opioid receptors (*μ*, *κ*, and *δ*), has garnered attention for its use in treating acute opioid overdose, managing alcohol dependence, and addressing addictive behaviors [7, 8]. Remarkably, nalmefene has demonstrated the ability to reverse postoperative opioid‐induced respiratory depression without compromising the analgesic response to subsequent opioid administrations [8, 9]. This dual action is potentially attributed to its antagonistic effects on *μ*‐receptors and partial agonistic effects on *κ*‐receptors in humans [10]. Given that the nausea and vomiting, respiratory depression, and addiction commonly associated with opioids are mediated through *μ*‐receptors, it stands to reason that nalmefene could potentially alleviate these adverse effects without undermining analgesia. Based on this theoretical framework, we embarked on a study to test the hypothesis that nalmefene can reduce opioid‐induced nausea and vomiting in the postoperative setting, thereby enhancing the overall safety and efficacy of PCA therapy.

## 1. Materials and Methods

**FIGURE 1:**
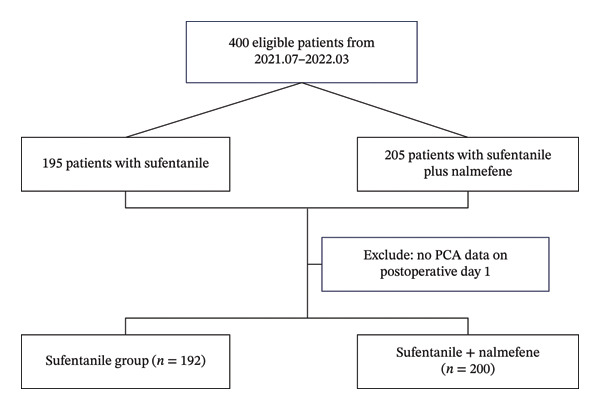
The consolidated standards of reporting trials statement. See text for details.

This study was a single‐center, retrospective cohort analysis conducted between July 2021 and March 2022, with the primary objective of investigating the incidence of nausea and vomiting and assessing the comparative effects of postoperative analgesia administered with sufentanil alone (sufentanil group) versus a combination of sufentanil and nalmefene (sufentanil + nalmefene group). The study protocol was rigorously reviewed and approved by the Ethics Committee of Zhongda Hospital of Southeast University, under the reference number 2022ZDSYLL440‐P01. Given the retrospective nature of the study, written informed consent was deemed unnecessary for all participants. The inclusion criteria were defined to encompass cases involving spine surgery patients who received postoperative PCA. Conversely, patients were excluded from the study if they lacked data on PCA initiation on postoperative Day (POD) 1 or if their PCA was discontinued for various reasons, including but not limited to transfer to the intensive care unit (ICU), the development of postoperative surgical complications, or personal refusal to continue PCA usage (Figure 1).

### 1.1. Data Source and PCA Setting

The data for this study were extracted from the electronic medical records within the surgical anesthesia information acquisition system and the Hospital Information System (HIS) of Zhongda Hospital of Southeast University. In the routine work of the department of anesthesiology, the evaluation of outcomes such as resting pain, activity pain, sedation score, nausea and vomiting, skin itching, urine retention, and flatus passage was assessed by a dedicated team of professionals who were blinded to the type of analgesic drugs administered through the PCA system. Postoperative follow‐ups were systematically conducted within 1–3 days after surgery, allowing for a comprehensive assessment of the patients’ recovery trajectory. The sufentanil group received a PCA formulation containing sufentanil (2 μg/kg) and tropisetron 6 mg, diluted to 150 mL. The sufentanil + nalmefene group received sufentanil (2 μg/kg), nalmefene (1 μg/kg), and tropisetron 6 mg, also diluted to 150 mL. The PCA settings, including the first dose, continuous dose, bolus dose, ultimate dose of 1 h, and lock time, were carefully documented. The lockout interval, defined as the period between the completion of one bolus administration and the subsequent allowance for another bolus, was maintained within a range of 10–20 min, ensuring a safe and effective delivery of analgesia. In cases where the attending ward doctor considered the analgesia inadequate, supplementary analgesia in the form of ketorolac tromethamine injection or flurbiprofen axetil injection was administered as needed. If patients developed PONV, rescue antiemetic treatment with tropisetron or azasetron was administered by ward physicians as part of routine clinical practice. This flexible approach to pain management underscores the commitment to optimizing patient outcomes and ensuring a seamless transition from surgery to recovery.

### 1.2. Various Scoring Criteria

The resting pain and activity pain were rigorously assessed using the Visual Analog Scale (VAS), a standardized tool with a range of 0–10, where 0 represents no pain, 1–3 signifies mild pain, 4–6 indicates moderate pain or intolerance, and 7–10 denotes severe pain that significantly disrupts sleep. Additionally, the Ramsay Sedation Score, ranging from 1 to 5, was employed to evaluate the level of sedation, with 1 indicating sobriety, 2 representing mild sleepiness, 3 signifying moderate sleepiness with ease of awakening, 4 indicating difficulty in awakening, and 5 corresponding to a state of nonawakening. The patient’s state of activity was meticulously scored on a 1‐4 scale, where 1 denotes movement restricted to the ground, 2 signifies movement requiring assistance, 3 indicates free movement within the bed, and 4 represents a complete inability to move within the bed. To comprehensively assess nausea and vomiting, a scoring system of 0–3 was utilized, with 0 representing the absence of nausea and vomiting, 1 indicating mild nausea, 2 signifying nausea and vomiting without the expulsion of stomach contents, and 3 denoting severe vomiting accompanied by the uncontrollable expulsion of stomach contents despite medication. This comprehensive approach ensures that all relevant aspects of the patient’s postoperative condition are thoroughly evaluated and documented.

### 1.3. Sample Size Calculation

The primary endpoint was the incidence of PONV within 24 h. Based on prior literature for sufentanil‐based PCA, we assume a control‐group PONV incidence of 28% and anticipate that the intervention will reduce this incidence to 15% (absolute difference 13%) [11]. Sample size was calculated for comparison of two independent proportions using a two‐sided test with *α* = 0.05 and power (1−β) = 0.80. The required sample size is 171 patients per group. To allow for an expected 15% loss to follow‐up or incomplete data, the per‐group sample size is increased to 200, yielding a total planned sample size of 400 patients. Ultimately, eight individuals were excluded based on the aforementioned exclusion criteria, resulting in a final study population of 392 patients.

### 1.4. Statistical Analysis

We analyzed independent variables using Student′s *t*‐test for normally distributed continuous variables (mean ± standard deviation [SD]) or the Wilcoxon rank‐sum test for abnormally distributed variables (median ± interquartile range). Categorical variables were presented as exact number and percentage of the total number of patients, and we used the chi‐square test or Fisher’s exact test. For the subgroup analysis, logistic regression was performed. All statistical analyses were performed utilizing the robust SPSS software, Version 16.0. Statistical significance was set at *p* < 0.05.

## 2. Results

The baseline characteristics and intraoperative data are depicted in Table 1. Sufentanil group and sufentanil + nalmefene group were similar in sex, body mass index (BMI), age, American Society of Anesthesiologists (ASA) classes, type of blood, type of insurance, and duration of surgery. The PCA setting details are depicted in Table 2. There were no differences between groups in the first dose, continuous dose, bolus dose, ultimate dose, and lock time.

**TABLE 1:** Demographic characteristics and intraoperative data.

	**Sufentanil group (*n* = 192)**	**Sufentanil + nalmefene group (*n* = 200)**	**p** **-value**

Age (y)	61.20 ± 11.87	61.47 ± 10.61	0.74
Sex *n* (%)	0.35		
Female	106 (55.21)	101 (50.50)	
Male	86 (44.79)	99 (49.50)	
Height (cm)	163.70 ± 7.15	164.82 ± 7.99	0.19
Weight (kg)	66.39 ± 10.94	67.45 ± 12.47	0.34
BMI	24.69 ± 3.18	24.75 ± 3.73	0.63
ASA physical status, *n* (%)	0.11		
I	12 (6.25)	7 (3.50)	
II	160 (83.33)	160 (80.00)	
III	20 (10.42)	33 (16.50)	
Duration of surgery (min)	174.99 ± 57.25	180.22 ± 63.81	0.39
Length of stay (day)	11.81 ± 4.47	11.76 ± 4.25	0.90
Cost of hospitalization (wan yuan)	10.02 ± 2.55	9.94 ± 2.97	0.78

*Note:* Data are presented as the mean ± SD or number of patients (%). ASA = American Society of Anesthesiologists.

Abbreviation: BMI = body mass index.

**TABLE 2:** PCA setting details.

	**Sufentanil group (*n* = 192)**	**Sufentanil + nalmefene group (*n* = 200)**	**p** **-value**

First dose (ug)	0 (0)	0 (0)	0.17
Continuous dose(ug)	0.67 ± 0.83	0.62 ± 0.52	0.48
Bolus does (ug)	1.55 ± 0.60	1.59 ± 0.61	0.67
Ultimate dose (ug)	6.69 ± 2.00	7.02 ± 2.16	0.07
Lock time (min)	15.96 ± 2.52	15.69 ± 2.16	0.10

*Note:* Data are presented as the mean ± SD or median (interquartile range).

Abbreviation: PCA = patient‐controlled analgesia.

The postoperative data are presented in Table 3. The primary endpoint was the incidence of PONV within 24 h postoperatively. The incidence of PONV was 15.5% (31/200) in the sufentanil + nalmefene group and 25.5% (49/192) in the sufentanil group. Rescue antiemetic use was significantly lower in the sufentanil + nalmefene group (6.0%, 12/200) compared with the sufentanil group (26.0%, 50/192). A statistically significant reduction in the rate of nausea and vomiting was observed on POD1 (*p* = 0.045), but not on POD2 or POD3 (*p* = 0.15 and *p* = 0.63, respectively). However, the VAS scores of resting pain were increased at the POD 2 (*p* = 0.04) but not POD 1 and 3 (*p* = 0.25 and *p* = 0.09). There were no differences between groups in the activity pain, Ramsay sedation score, state of activity, and the percentage of flatus passage at POD 1, 2, and 3. Considering that PONV is influenced by multiple well‐established factors, such as duration of surgery, sex, and ASA physical status, we performed a logistic regression analysis to evaluate the effect of nalmefene across different patient characteristics (sex, age, BMI, ASA physical status, and duration of surgery). Sex was identified as a significant factor (*p* < 0.01); therefore, we conducted a subgroup analysis by sex (Table 4). The analysis revealed that nalmefene significantly reduced nausea and vomiting scores in male patients (*p* = 0.04). As shown in Table 5, there were also no differences between groups in blood inflammation markers.

**TABLE 3:** Postoperative data.

	Sufentanil group	Sufentanil + nalmefene group	*p*‐value
POD1	*n* = 192	*n* = 200	
Resting pain score	1.39 ± 1.31	1.54 ± 1.36	0.25
Activity pain score	1.90 ± 1.47	2.14 ± 1.49	0.11
Ramsay sedation score	1.01 ± 0.13	1.01 ± 0.14	0.72
State of activity score	3.64 ± 0.51	3.72 ± 0.51	0.13
Nausea and vomiting score	0.34 ± 0.65	0.22 ± 0.56	0.045^∗^
Flatus passage (%)	0.00	0.00	1.00
POD2	*n *= 192	*n *= 197	
Resting pain score	1.34 ± 1.19	1.60 ± 1.35	0.04^∗^
Activity pain score	2.89 + 1.12	2.94 + 1.10	0.62
Ramsay sedation score	1.01 + 0.14	1.00 + 0.01	0.32
State of activity score	3.05 + 0.34	3.07 + 0.28	0.66
Nausea and vomiting score	0.11 + 0.42	0.06 + 0.30	0.15
Flatus passage (%)	62.50	57.70	0.35
POD3	*n* = 166	*n* = 172	
Resting pain score	1.10 + 1.09	1.31 ± 1.32	0.09
Activity pain score	2.57 + 1.09	2.80 ± 1.77	0.18
Ramsay sedation score	1.01 + 0.16	1.02 ± 0.17	0.76
State of activity score	3.03 + 0.17	2.98 ± 0.37	0.13
Nausea and vomiting score	0.03 + 0.28	0.02 ± 0.23	0.63
Flatus passage (%)	70.90	64.30	0.20

*Note:* Data are presented as the mean ± SD or number of patients (%), and *p*‐value < 0.05 is marked with an asterisk (^∗^). POD, postoperative day.

**TABLE 4:** Subgruop analysis of nausea and vomiting score by gender.

**Gender**	**Group**	***n***	**The score of nausea and vomiting**	**p** **-value**

Male	Sufentanil	86	0.20 ± 0.55	0.04^∗^
	Sufentanil + nalmefene	99	0.06 ± 0.34	

Female	Sufentanil	106	0.45 ± 0.71	0.37
	Sufentanil + nalmefene	101	0.37 ± 0.67	

*Note:* Data are presented as the mean ± SD or number of patients (%), *p*‐value < 0.05 is marked with an asterisk (^∗^).

**TABLE 5:** Preoperative and postoperative blood inflammation levels.

	**Sufentanil group (*n *= 192)**	**Sufentanil + nalmefene group (*n *= 200)**	**p** **-value**

*Perioperative*			
WBC (10^9/L)	6.05 + 1.82	5.76 ± 1.62	0.09
Neutrophils (%)	58.67 + 9.99	59.02 ± 9.81	0.73
ESR (mm/h)	11.88 + 13.67	11.23 ± 10.97	0.61
C‐P (mg/L)	3.84 + 7.33	3.68 ± 7.91	0.84

*Postoperative*			
WBC (10^9^/L)	8.09 + 2.58	7.99 ± 2.32	0.66
Neutrophils (%)	72.58 + 8.76	71.29 ± 9.41	0.16
ESR (mm/h)	44.42 + 26.14	42.42 ± 24.52	0.51
C‐P (mg/L)	38.83 + 35.17	37.22 ± 36.28	0.68

*Note:* Data are presented as the mean ± SD or number of patients (%). WBC, white blood cell count; C‐P, C‐reactive protein.

Abbreviation: ESR = erythrocyte sedimentation rate.

## 3. Discussion

This retrospective investigation revealed that while nalmefene effectively mitigated nausea and vomiting induced by sufentanil analgesia, it notably elevates patients’ resting pain intensity, with no discernible impact on movement‐related pain, flatus passage, activity levels, or sedation status. Although nalmefene has been reported to attenuate inflammatory responses, the present study did not demonstrate a reduction in inflammatory markers in the nalmefene group [12–14].

Postoperative pain represents a pervasive challenge in the postoperative phase, and the provision of efficacious analgesia is paramount to minimizing adverse postoperative outcomes. Since the 1960s, PCA pumps have emerged as a prevalent modality for alleviating postoperative pain, offering patients greater autonomy over their pain management [15]. These devices expedite the response to pain by minimizing the delay between pain perception and medication administration, enhancing pain control without imposing an undue burden on nursing staff. In this study, postoperative resting pain gradually ameliorated over time, whereas movement‐induced pain significantly intensified as patients embarked on postoperative mobilization, typically commencing on the second day postsurgery. Notably, the addition of nalmefene to PCA pumps was associated with slightly higher postoperative pain scores, with a statistically significant increase in resting pain observed on POD 2. One possible explanation is that nalmefene, as an opioid receptor modulator, may interact with opioid‐mediated analgesia when coadministered in PCA pumps. However, this difference was limited to resting pain on POD 2, a period when patients typically begin to increase mobilization. Although activity‐related pain scores were numerically higher in the nalmefene group, the difference did not reach statistical significance. Therefore, the balance between its potential impact on analgesia and its antiemetic benefit should be considered on an individual patient basis in clinical practice.

PONV are predominantly triggered by inhalational anesthetics and opioid analgesics, with youth, female gender, nonsmoking status, and a history of motion sickness being key contributing factors. Despite the implementation of a triple prophylactic regimen encompassing dexamethasone, ondansetron, and transdermal scopolamine (TDS), the incidence of PONV remains high following general anesthesia, with studies indicating that up to 42.7% of bariatric surgery patients necessitate rescue antiemetics [15]. Opioid‐induced nausea and vomiting stem primarily from the activation of *μ*‐receptors, often constraining the utilization of PCA pumps and, in some instances, leading to patient refusal due to these adverse effects [16]. Our study underscores the efficacy of nalmefene in mitigating PONV in patients over the initial three PODs; especially in the first day, the incidence of PONV was significantly decreased 10.02% (25.52% in the sufentanil group vs 15.50% in the sufentanil + nalmefene group). The observation aligns with the significantly lower use of rescue antiemetics in the sufentanil + nalmefene group on the first POD. Although some studies have reported that nalmefene may activate the hypothalamic–pituitary–adrenal (HPA) axis, thereby increasing circulating cortisol levels and modulating inflammatory responses, which could contribute to the suppression of nausea and vomiting, no significant differences in inflammatory biomarkers were observed between the two groups in our study. This suggests that the antiemetic effect of nalmefene may not be primarily mediated through systemic anti‐inflammatory pathways [17, 18]. Instead, its effects may be related to neuroendocrine modulation. Nalmefene is an opioid receptor modulator that antagonizes *μ*‐opioid receptors and partially activates *κ*‐opioid receptors, potentially influencing nausea and vomiting through central neuromodulatory mechanisms involving the chemoreceptor trigger zone and the vomiting center in the brainstem. In our subgroup analysis, we found that nalmefene significantly reduced the score of PONV in male patients but not in female patients. One possible explanation for this sex‐specific difference is that women inherently have a higher baseline risk of PONV, which may make them relatively less sensitive to the antiemetic effect of nalmefene. In addition, sex‐related differences in hormonal status, opioid sensitivity, and pharmacodynamic responses may also contribute to variability in antiemetic efficacy. As this study was retrospective and not specifically powered for detailed subgroup analyses, these findings should be interpreted with caution and require confirmation in future prospective studies.

However, it is important to acknowledge the limitations of our study. First, our study was a single‐center retrospective analysis. To validate our findings and achieve a more comprehensive understanding, future research should involve multicenter prospective randomized clinical trials. Second, the anticipated decrease in white blood cell count among patients in the nalmefene group was not observed, which could be attributed to the relatively modest sample size. Furthermore, several clinically relevant confounders—including additional intraoperative antiemetic use, patient comorbidities, intraoperative opioid consumption, and variations in surgical or anesthetic techniques—were not fully adjusted for. Given the retrospective nature of the study and the limited availability of these variables, residual confounding cannot be excluded and may have influenced the results. Finally, although the difference in PONV scores on POD 1 reached statistical significance, the absolute mean difference of 0.12 on our 4‐point ordinal scale (0–3) is small and likely of limited clinical relevance. Nevertheless, the use of rescue antiemetics was clearly lower in the sufentanil + nalmefene group, indicating that nalmefene may have a meaningful impact on PONV. No minimally clinically important difference (MCID) has been established for this scale, and previous studies using broader PONV measures (e.g., 0–50) provide context for clinically meaningful changes [19]. Taken together, more sensitive and validated PONV assessment tools, as well as appropriate patient‐centered endpoints, are needed to better evaluate the clinical significance of antiemetic interventions.

In summary, despite the finding that nalmefene infusion into PCA pumps may marginally elevate postoperative resting pain levels, it does not significantly impact movement pain and is highly effective in significantly reducing the incidence of PONV in patients. This retrospective study underscores the potential of nalmefene as a valuable adjunct in postoperative analgesia.

## Author Contributions

Jiaojiao Yang and Baoling Zhang designed the study, and Jiaojiao Yang authored the manuscript. Data analysis was conducted by Lili Qiu and Baoling Zhang. Jue Xie and Qiuting Zeng contributed to the study execution, and Jie Sun edited the manuscript. The manuscript was reviewed by all authors.

## Funding

This work was supported by grants from the National Natural Science Foundation of China (Grant Nos. 81801074 and 82201425).

## Ethics Statement

The Ethics Committee of Zhongda Hospital, affiliated with Southeast University, has given its approval for this study, documented under the approval number No. 2022ZDSYLL440‐P01. Given the retrospective nature of the study, written informed consent was deemed unnecessary for all participants.

## Conflicts of Interest

The authors declare no conflicts of interest.

## Data Availability

The data that support the findings of this study are available from the corresponding author upon reasonable request.
